# Test for Arteriosclerosis

**Published:** 1914-06

**Authors:** 


**Test for Arteriosclerosis.** Hertzell, Berliner Klin. Woch., No. 6, 1913, places the patient in bed and shuts off the circulation in one arm and both legs. Normally, there will be an increase in blood pressure of only 5 millimeters. In arteriosclerosis, owing to inelasticity of arteries, it may amount to 60 or more.

